# On-chip distribution of quantum information using traveling phonons

**DOI:** 10.1126/sciadv.add2811

**Published:** 2022-11-18

**Authors:** Amirparsa Zivari, Niccolò Fiaschi, Roel Burgwal, Ewold Verhagen, Robert Stockill, Simon Gröblacher

**Affiliations:** ^1^ Kavli Institute of Nanoscience, Department of Quantum Nanoscience, Delft University of Technology, 2628CJ Delft, the Netherlands.; ^2^ Center for Nanophotonics, AMOLF, Science Park 104, 1098XG Amsterdam, the Netherlands.; ^3^ Department of Applied Physics, Eindhoven University of Technology, P.O. Box 513, 5600MB Eindhoven, the Netherlands.

## Abstract

Distributing quantum entanglement on a chip is a crucial step toward realizing scalable quantum processors. Using traveling phonons—quantized guided mechanical wave packets—as a medium to transmit quantum states is now gaining substantial attention due to their small size and low propagation speed compared to other carriers, such as electrons or photons. Moreover, phonons are highly promising candidates to connect heterogeneous quantum systems on a chip, such as microwave and optical photons for long-distance transmission of quantum states via optical fibers. Here, we experimentally demonstrate the feasibility of distributing quantum information using phonons by realizing quantum entanglement between two traveling phonons and creating a time-bin–encoded traveling phononic qubit. The mechanical quantum state is generated in an optomechanical cavity and then launched into a phononic waveguide in which it propagates for around 200 micrometers. We further show how the phononic, together with a photonic qubit, can be used to violate a Bell-type inequality.

## Teaser

A platform for traveling phonons on a chip is demonstrated, which will enable hybrid quantum devices and networks.

## INTRODUCTION

Over the past decades, quantum technologies have evolved from scientific proof-of-principle experiments to a nascent and thriving industry. With recent demonstrations of quantum advantage over classical computation in multiple systems (*1*, *2*), the need for connecting these resources is becoming ever more urgent. Distributing quantum entanglement between distant parties is a crucial step toward implementing quantum repeaters and networks (*3*, *4*). Distributing it on-chip is needed for sparse qubit array architectures, which require on-chip long-range qubit couplers (*5*). In addition, having entanglement between a stationary quantum memory and a flying qubit plays a central role in low-loss quantum information transfer over long distances (*3*).

One of the key challenges of building a quantum network is forming interfaces between heterogeneous quantum devices. A highly versatile system for this task has been identified in phonons, which can act as efficient intermediaries between different resources (*6*). In particular, phonons have been shown to be highly useful in converting states between different optical wavelengths (*7*), as well as for microwave-to-optics frequency conversion (*8*–*14*). Most recently, such a mechanical transducer has been used to transfer signals from a superconducting qubit to an optical fiber (*15*), a key step for quantum information transfer. Moreover, the potential for quantum gate operations using phonons has been shown (*16*–*18*), owing to long coherence times and high transfer fidelities of phonons. The interest in traveling phonons goes well beyond enabling long-distance quantum networks. Several of the most exciting prospects are arising from their many orders of magnitude slower propagation speed compared to light, low-loss transmission and their small mode volume compared to traveling Gigahertz photons. These unique features could have the potential to enable the on-chip distribution and processing of quantum information in a highly compact fashion (*19*), allowing for coherent interactions with a large variety of quantum systems such as defect centers (*20*), superconducting qubits (*21*), and quantum dots (*22*, *23*), in both homogeneous or heterogeneous implementations (*24*). Demonstrating the basic building blocks, such as marking the distribution of quantum information using highly confined phonons, remains an open challenge to date.

Quantum optomechanics has proven to be a versatile toolbox for controlling stationary, strongly confined phonons (*25*, *26*). Previously, bulk and surface acoustic waves (BAWs and SAWs, respectively) have been shown to be able to operate in the quantum regime (*17*, *19*), for example, by coupling to superconducting qubits for transducer and quantum information applications (*27*, *28*), as well as entangling acoustic phonons (*29*). These systems benefit from deterministic quantum operations with high fidelities, enabled by the nonlinearity of the superconducting qubit and strong coupling between the qubit and the phononic channel. However, the confinement of the phonons in optomechanical devices results in several advantages, such as stronger field coupling (*30*), higher coherence and longer lifetime (*31*), and long-distance routing capability on chip (*32*, *33*). In this work, building on these recent developments, we experimentally distribute quantum information using phonons in a waveguide. Our device is composed of an optomechanical cavity, which acts as the single-phonon source and detector, that is connected to a single-mode phononic waveguide. Using this device, we then create a time-bin–entangled state of a pair of traveling phonons. Furthermore, we unambiguously show the nonclassical correlations between an optical and the traveling phononic qubit by violating a Bell-type inequality (*34*, *35*).

## RESULTS

**Fig. 1.**
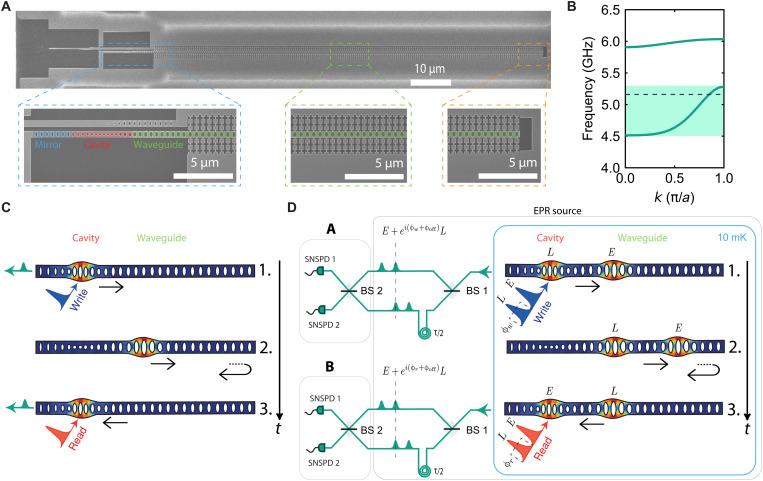
Device and experimental setup. (**A**) Scanning electron microscopy image of the device. Bottom left: Photonic and phononic mirror (highlighted in blue), optomechanical cavity (red), and initial part of the phononic waveguide, which also acts as a photonic mirror (green). Bottom center: Section of the phononic waveguide. Bottom right: Free-standing end of the waveguide, which acts as a mirror for the phonons. (**B**) Band diagram of a unit cell of the waveguide showing its single-mode design for the symmetric mode, with the frequency of interest depicted by the black dashed line (see section S1). (**C**) Control pulses (write and read) are sent to the cavity to create (1.) and retrieve (3.) the mechanical excitation. The green pulses depict the scattered Stokes and anti-Stokes photons. In (2.), the mechanical excitation travels in the waveguide (round-trip time of τ). (**D**) Simplified schematics of the time-bin entangling protocol. (1.) Creation of the entangled state between the Stokes-scattered photons and the traveling phononic excitation in the waveguide. The pulses have a time delay of τ/2 and are depicted here in shorter succession than in the experiment for clarity of the drawing. (2.) Propagation of the mechanical qubit in the waveguide, with the reflection at the end. (3.) Mapping of the phononic onto a photonic state to verify the entanglement. The boxes conceptually divide Einstein-Podolsky-Rosen (EPR) source, and measurements A and B (which are the same experimental setup at different times *t*), used to create and detect the entangled state. SNSPDs are superconducting nanowire single-photon detectors. The arrows in (C) and (D) represent the direction of propagation of the mechanical excitations, and the time axis is shown by the vertical black arrow.

Our device consists of a single-mode optomechanical cavity connected to a phononic waveguide (cf. Fig. 1A), similar to a previous design (*33*). The cavity is used as a source and detector for mechanical excitations, controlled with telecom-wavelength optical pulses [via Stokes and anti-Stokes scattering (*36*)]. We engineer the photonic and phononic band structure of the different parts of the device such that the mechanical mode extends into the waveguide while the optical mode remains fully confined in the cavity (see the Supplementary Materials for more details on the device design). The waveguide has a free-standing end, which acts as mirror for the traveling phonons and effectively forms a Fabry-Pérot cavity. The coupling between the single-mode cavity and this Fabry-Pérot cavity results in a hybridization of the cavity and waveguide modes into (almost) evenly spaced modes separated by the free spectral range (FSR) of the Fabry-Pérot cavity. The FSR is determined by the length of the waveguide and by the group velocity of the phonons. We design the waveguide to be single mode for the symmetry of the mechanical mode used in this work (the band structure is shown in Fig. 1B).

To create a propagating mechanical excitation, we use a blue-detuned write (Stokes) control pulse, which, via a two-mode squeezing interaction, creates entangled photon-phonon pairs. The phononic excitation created in the cavity then leaks into the waveguide, is reflected by the end mirror, and returns back periodically to the cavity after a round-trip time τ. Last, to retrieve the mechanical state, a red-detuned read (anti-Stokes) control pulse enables the optomechanical beam-splitter interaction that maps the mechanical into a photonic excitation (see Fig. 1C for details on the scheme). To create a time-bin–encoded mechanical qubit using this scheme, we first place the device in a dilution refrigerator at 10 mK, initializing the mechanical mode in its quantum ground state. We then send two blue-detuned write pulses separated by τ/2 to the device in the cryostat, as shown in Fig. 1D, and send the resulting scattered photons into an optical interferometer. One arm is delayed with respect to the other one by τ/2 to overlap the scattered photons in time. The reflected control pulses are suppressed using optical filters (see the Supplementary Materials), and the resulting interferometer output signals are detected on two superconducting nanowire single-photon detectors (SNSPDs). By operating in the low pulse energy regime (low optomechanical scattering probabilities, *p*_w_), the two identical write pulses create the optomechanical state

$$|\psi_{0}\rangle \propto |0000\rangle +\sqrt{p_{w}}({|1010\rangle }_{E_{o}L_{o}E_{m}L_{m}}+e^{i\phi_{w}}{|0101\rangle }_{E_{o}L_{o}E_{m}L_{m}})+O(p_{w})$$ (1)

where *E*_m_ (*L*_m_) and *E*_o_ (*L*_o_) indicate the “early” (“late”) mechanical and optical states, respectively, and ϕ_w_ is the phase difference between the two early and late write pulses, set with an electro-optical modulator (see section S4 for more details). By overlapping these early and late photons on a beam splitter, after passing through the unbalanced interferometer, we erase any “which path” information. Consequently, by detecting a Stokes-scattered photon from the overlapped early and late pulses on one of the detectors, we perform an entanglement swapping operation, resulting in an heralded entangled state between the early and late traveling mechanical excitations

$$|\psi_{m}\rangle \propto {|10\rangle }_{E_{m}L_{m}}\pm e^{i(\phi_{w}+\phi_{\text{off}})}{|01\rangle }_{E_{m}L_{m}}$$ (2)

with the plus (minus) sign resulting from a detection event in either detector. The phase ϕ_off_ is a fixed phase difference between the two arms of the unbalanced interferometer (sections S5 and S6). Note how the state is maximally entangled in the Fock basis, which at the same time serves as a mechanical qubit.

The entangled phonon state travels through the waveguide and, after reentering the cavity, can be mapped onto an optical state with the red-detuned anti-Stokes control pulses. The entire four-mode optical state of write and read scattered photons can be expressed as

$$\begin{array}{c} |\psi_{\mathrm{AB}}\rangle \propto [(1+e^{i(\phi_{w}+\phi_{r}+2\phi_{\text{off}})})({\hat{a}}_{w,1}^{†}{\hat{a}}_{r,1}^{†}-{\hat{a}}_{w,2}^{†}{\hat{a}}_{r,2}^{†})+\\ i(1-e^{i(\phi_{w}+\phi_{r}+2\phi_{\text{off}})})({\hat{a}}_{w,2}^{†}{\hat{a}}_{r,1}^{†}+{\hat{a}}_{w,1}^{†}{\hat{a}}_{r,2}^{†})]|0000\rangle \end{array}$$ (3)

where ${\hat{a}}_{w,1}^{†}.$ (${\hat{a}}_{w,2}^{†}$) and ${\hat{a}}_{r,1}^{†}$ (${\hat{a}}_{r,2}^{†}$) are the creation operators of the photon coming from the write pulse and read pulse, respectively, on detector 1 (detector 2). An additional phase ϕ_r_ is applied only on the late read pulse, which is used to rotate the readout basis. Here, the read pulses map the mechanical state onto the optical mode, and hence, this state is a direct result of the entanglement between the photonic and the traveling phononic qubit. For verifying the mechanical entanglement of Eq. 2, we use ϕ_r_ = 0.

In order for our protocol to work, we need to fulfill several basic requirements. For both the phononic and photonic qubits, for example, we have to create orthogonal states (the basis), and thus, for the time-bin encoding, we have to be able to unambiguously distinguish the early and late states. Experimentally, we implement this by realizing a ∼100-μm-long phononic waveguide and by choosing the control pulse length as 30 ns, given a simulated group velocity in the waveguide of approximately 2000m/s. Moreover, the thermal occupation of the mechanical mode, mainly given by a small absorption of the control pulses in the optomechanical cavity, has to be ≪1 to realize a high-fidelity entangled state. This limits the maximum scattering probability of the write and read control pulses (see section S3).

**Fig. 2.**
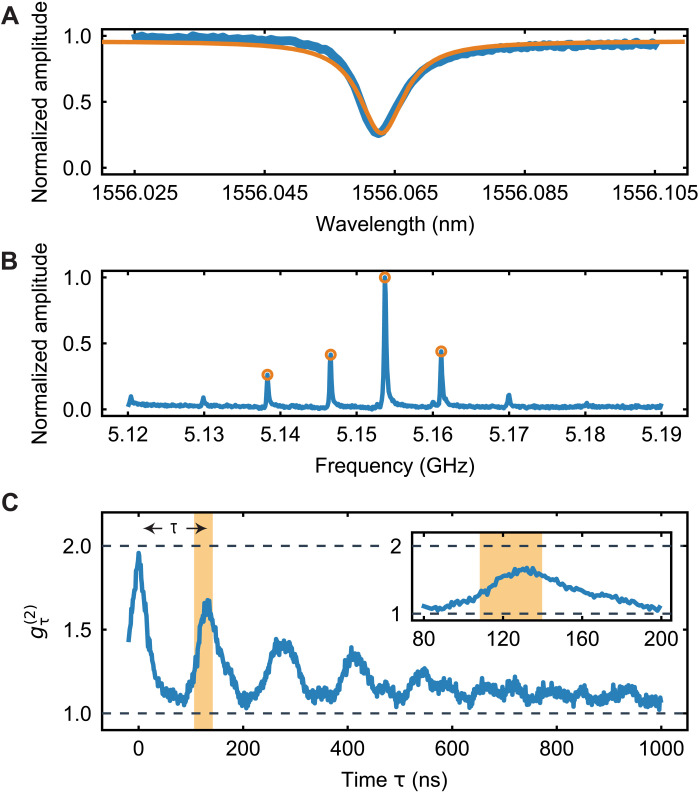
Initial characterization. (**A**) Optical spectrum of the device measured in reflection. (**B**) Mechanical spectrum measured using optomechanically induced transparency. (**C**) Second-order correlations of a thermal state for different time delays between the SNSPDs click events τ. The series of equally spaced peaks shows that, when the thermal mechanical excitations leave the optomechanical cavity, they are reflected from the end of the waveguide and then return back into the cavity. The inset is a close-up of the area around the first peak. The shaded regions show the delay and control pulse area chosen for all the subsequent experiments in this work (τ = 126 ns, with a time length of 30 ns).

To characterize the device, we first measure its optical properties at 10 mK and observe a resonance at λ ≈ 1556.06 nm with full width at half maximum (FWHM) of κ/2π ≈ 1.05 GHz (intrinsic loss rate κ_i_/2π = 250 MHz; see Fig. 2A). We use the optomechanically induced transparency technique to measure the mechanical spectrum of the device (*33*, *37*). As can be seen in Fig. 2B, the hybridized modes exhibit a clean, evenly spaced spectrum with FSR ≈ 8 MHz. We choose the most prominent mechanical resonance in Fig. 2B (around 5.154 GHz) as the frequency to which we detune the lasers with respect to the optical resonance to address Stokes and anti-Stokes interactions. We further use the rate of Stokes-scattered photons from a 30-ns-long pulse to determine the equivalent single-photon optomechanical coupling rate (*38*) of the ensemble of optomechanically coupled modes at *g*_0_/2π ≈ 380 kHz.

To measure the round-trip time and coupling between the cavity and waveguide, we pump our device with a continuous red-detuned laser. Because of the nonzero optical absorption in the device, the continuous laser creates a thermal mechanical population inside the optomechanical device that leaks into the phononic waveguide and reflects from the free-standing end before returning back into the optomechanical cavity. The same red-detuned laser then maps the mechanical state onto a photonic state, and we measure the two-photon detection coincidence with varying delays between two events. This measurement allows us to obtain the intensity correlation $g_{\tau }^{\left(2\right)}$ of the mechanical thermal state in the optomechanical cavity, as can be seen in Fig. 2C. The coincidence rate is normalized to the single-photon click rates. For zero time delay, we find a $g_{\tau =0}^{\left(2\right)}\approx 2$, as expected for a thermal state. The correlation drops down to 1, as the thermal state leaves the optomechanical cavity into the waveguide, resulting in uncorrelated clicks, and then periodically increases again when the thermal population returns back to the cavity (*33*). We use this measurement to determine the round-trip time for an excitation in the waveguide, τ= 126 ns. Note that the decay in the peak values is mainly due to the unequal small difference in FSR between the mechanical modes (*33*) and the short mechanical lifetime *T*_1_ ≈ 2.2 μs (see section S7 for more details). From the FWHM of the peak centered around zero time delay in Fig. 2C, we can extract the packet time duration of ≈30 ns. To match the packet time length, we then choose to use 30-ns-long Gaussian write and read pulses in all experiments.

**Fig. 3.**
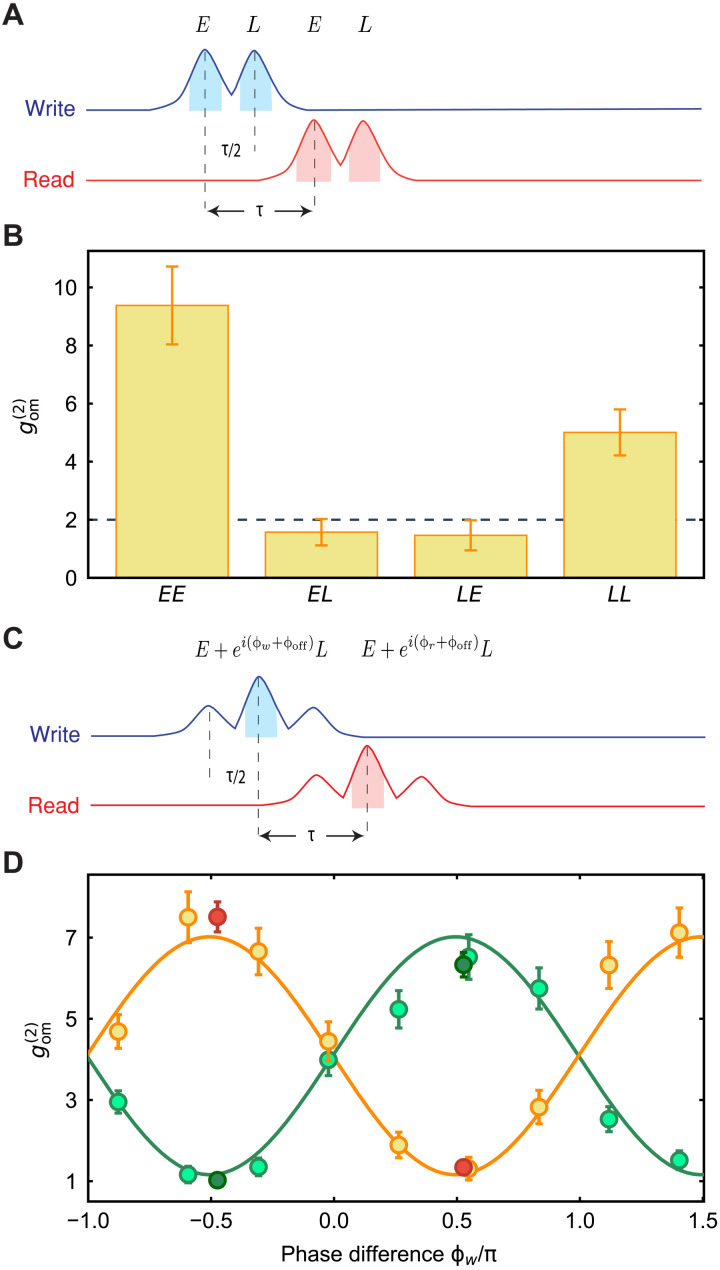
Time-bin phononic entanglement. (**A**) Pulse scheme for a double write/read pulse cross-correlation measurement. In this experiment, the delayed arm of the interferometer is open. The two write and two read pulses are called early (*E*) and late (*L*) and are delayed by τ/2. The shaded areas are the regions from which the coincidences are gathered (time length of 30 ns). (**B**) Extracted values of the cross-correlation $g_{\text{om}}^{\left(2\right)}$ measured for the four combinations of write/read pulses. The correlations for *EE* and *LL* are significantly exceeding the classical threshold of 2 (dashed line), while the other two combinations of *EL* and *LE* only exhibit classical correlations. (**C**) Control pulse scheme to create and detect time-bin phononic entanglement at the interferometer. The early pulse passing through the delay arm of the interferometer and the late pulse passing through the direct arm of the interferometer overlap in time. (**D**) Second-order correlations of the Stokes and anti-Stokes photons as a function of the relative phase difference ϕ_w_ between the early and late write pulses. The events for same detector coincidences are shown in green, while different detectors coincidences are orange. Two additional measurements, red and dark green points, are performed at two phase settings to obtain more statistics for verifying the phononic entanglement. The maximum violation is *R* = 0.72 ± 0.06, almost 5 SDs below the classical threshold of 1. All error bars are 1 SD. The solid curves are the joint fit of the data and serve as guide to the eye.

As a next step, we verify our ability to distinguish between multiple-phonon wave packets. To do this, we measure the photon cross-correlations in a double write/read pulse sequence, in which we create and measure the second wave packet after half of the round-trip time τ/2 ≈ 63 ns. In this experiment, the delay arm of the interferometer is disconnected, such that the pulses do not interfere. Two write and two read pulses are sent to the device with a relative delay between them of τ/2, as shown in Fig. 3A. In all the pulsed measurements, we set a waiting time between trials of ∼7 *T*_1_ (15 μs) to let the mechanical modes thermalize to the ground state (see section S3). The energy of each pulse is 26 fJ (112 fJ) for the write (read), a probabilistically scattering photons through the Stokes (anti-Stokes) process, with a probability of *p*_w_ = 0.2% (*p*_r_ = 0.7%). The measured thermal phonon numbers of the mechanical resonator after applying the four pulses are 0.022 ± 0.002, 0.040 ± 0.003, 0.066 ± 0.003, and 0.095 ± 0.004 (cf. section S3). We measure the second-order cross-correlation between the four combinations of early and late write and read pulses, $g_{\text{cc}}^{\left(2\right)}$ as shown in Fig. 3B. We observe strong nonclassical correlations of $g_{\text{cc}}^{\left(2\right)}=9.4\pm 1.3$ between early-early and $g_{\text{cc}}^{\left(2\right)}=5.0\pm 0.8$ between late-late combinations, while the other two combinations show only classical correlations of $g_{\text{cc}}^{\left(2\right)}=1.5\pm 0.5$ (*39*). Note that the lower value for the late-late combination, with respect to early-early, is caused by the small accumulated thermal population induced by the pulses (see section S3 for more information).

We now proceed to verify that we have created a traveling mechanical qubit encoded in a superposition of early and late time bins, by sending the same pulse sequence to the device, with the delay arm of the interferometer connected. This way, the part of the early scattered photons that pass through the delay line and the part of the late scattered photons that pass through the direct arm are overlapped in time, such that a single-photon detection event after BS2 projects the mechanical state in Eq. 2. The pulse sequence at the detectors is shown in Fig. 3C, where the highlighted peaks are the overlapped and interfered early and late pulses, from which we detect the photons. We sweep the excitation phase ϕ_w_ and measure the second-order correlation *g*^(2)^ between the write and read photon detection events occurring at the same (green) or different (orange) output of BS2, displayed in Fig. 3D. The periodic dependence on the phase demonstrates the coherence of the generated entangled state. To show that the state shown in Eq. 2 is entangled, we use an entanglement witness, denoted by *R*, designed for optomechanical systems (*40*), as previously used in (*41*). After sufficient integration, we gather more than 500 coincidence events, and we obtain *R* = 0.72 ± 0.06, violating the classical threshold of 1 by almost 5 SDs.

To unambiguously demonstrate the nonclassical character of the traveling phononic qubit and the photon state in Eq. 3, we perform a Bell-type test using the Clauser-Horne-Shimony-Holt (CHSH) inequality (*34*). We define the correlation coefficients

$$E(\phi_{w},\phi_{r})=\frac{n_{11}+n_{22}-n_{12}-n_{21}}{n_{11}+n_{22}+n_{12}+n_{21}}$$ (4)

where *n_kl_* are the events where detector *k* clicked after a write pulse (station A in Fig. 1D) and detector *l* after a read pulse (station B in Fig. 1D). The inequality then states that

$$S=|E(\phi_{w}^{0},\phi_{r}^{0})-E(\phi_{w}^{1},\phi_{r}^{0})+E(\phi_{w}^{0},\phi_{r}^{1})+E(\phi_{w}^{1},\phi_{r}^{1})|\leq 2$$ (5)

The maximum violation is expected to occur for [$\phi_{w}^{i}=\phi_{0}+{\left(-1\right)}^{i+j}\pi /4$, $\phi_{r}^{j}={\left(\pi /2\right)}^{j}$], with *i*, *j* = {0,1}, and where ϕ_0_ = 2ϕ_off_ + π/2 ≈1.0π is the phase for which the correlation coefficient is zero (with negative slope). We choose phase settings with a small offset compared to these values to have the highest possible value of *S* for our setup (see section S6 for more details). To violate the CHSH inequality, we lower the energy of the write pulses slightly, such that we use 15 fJ (112 fJ) for the write (read) pulse, with a scattering probability of *p*_w_ = 0.13% (*p*_r_ = 0.7%). The measured thermal populations for the four pulses are then 0.027 ± 0.003, 0.038 ± 0.004, 0.055 ± 0.002, and 0.090 ± 0.004 (see section S3).

**Fig. 4.**
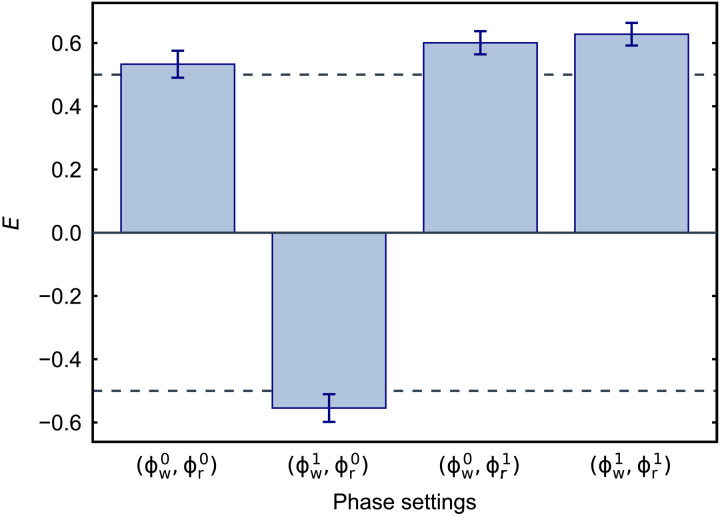
Bell test. The values of the correlation coefficients *E* grouped for the four ideal phase settings of the CHSH inequality, which are $(\phi_{w}^{0},\phi_{r}^{0})=(\phi_{0}-\pi /4$, 0), $(\phi_{w}^{1},\phi_{r}^{0})=(\phi_{0}+\pi /4$, 0), $(\phi_{w}^{0},\phi_{r}^{1})=(\phi_{0}-\pi /4$, π/2), and $(\phi_{w}^{1},\phi_{r}^{1})=(\phi_{0}+\pi /4$, π/2). The total number of events for each phase setting is ∼400. The dashed lines are the threshold for each correlation coefficient to violate the inequality. From these values, we obtain *S* = 2.32 ± 0.08, which violates the inequality by 4 SDs. All errors are 1 SD.

From the correlation coefficient, we define the visibility as *V* = max (∣*E*∣). We first perform an additional measurement at the phase of maximum visibility obtaining *V* = 0.82 ± 0.04, which is considerably higher than the threshold of $V>1/\sqrt{2}\approx 0.7$ required for violating the CHSH inequality. We then measure at the four optimal phase settings for the Bell test (see Fig. 4) obtaining a value of *S* = 2.32 ± 0.08, which corresponds to a violation of the CHSH inequality by 4 SDs. The rate of events for these measurements is around 30 per hour of integration, allowing us to measure the full dataset for the violation within 56 hours.

## DISCUSSION

We have unambiguously demonstrated a traveling phononic qubit in the form of a time-bin–entangled state, which can be used to distribute quantum information on a chip. The routing process is shown to be fully coherent, which is of fundamental importance for routing quantum information and interconnecting quantum devices. While we limit ourselves to show two-mode entanglement, the same device can be used with up to four modes, given the round-trip time and mechanical packet length, or more by using a longer waveguide. In addition, the quantum state can be retrieved at arbitrary multiples of the round-trip time, allowing for long storage and controlled emission of the state. Moreover, as the phononic entangled state travels down the waveguide, a straightforward extension using our device will allow distributing the quantum entanglement to different points on a chip. We have chosen to use a waveguide design with a lifetime of only *T*_1_ ≈ 2.2 μs, limiting the maximal phonon traveling length for this device to around 3 mm. By adding additional phononic shielding, this can, however, easily be extended to meter scales as the device’s lifetime increases to several milliseconds (*31*).

The demonstrated time-bin entanglement between a photonic and a traveling phononic qubit, verifying their nonclassical correlations by violating a CHSH inequality, underlines the suitability of the phononic system as a Duan-Lukin-Cirac-Zoller (DLCZ) unit cell (*42*). In this work, the fidelity of the entangled state is limited by residual optical absorption, which can be further reduced by up to an order of magnitude through optimized fabrication, allowing for state retrieval efficiencies of up to 30% (*39*).

The ability to excite, guide, and detect traveling phonons is the basic toolbox for phonon manipulation on-chip, enabling a completely new field using traveling mechanical modes in the quantum regime. Together with a phononic phase modulator (*43*) and beam splitter, this work will lead to full coherent control of guided phonons and paves the way to advanced quantum acoustic experiments. Moreover, our measurements highlight the potential of phonons as ideal candidates for realizing quantum networks and repeaters, as well as for on-chip distribution of quantum information in hybrid quantum devices, for example, for interfacing microwave superconducting circuits with spin quantum memories (*24*) or to couple on-chip qubits using electron-phonon interaction in solids (*22*).

## METHODS

The device is fabricated from a silicon-on-insulator wafer with a 250-nm-thick silicon device layer. We use electron beam lithography to pattern the structure and transfer the mask with a dry HBr/Ar plasma etch. The chip is diced to access the device’s optical waveguide with a lensed fiber in the dilution refrigerator. Last, the device is cleaned with a piranha solution and released using a 40% hydrofluoric acid (HF) wet etch to remove the oxide layer (*36*). After room temperature characterization and just before the cooldown in the cryostat, we perform another piranha cleaning and a 1% HF dip to minimize the native oxide.

## Supplementary Materials

### This PDF file includes:

Sections S1 to S7 Figs. S1 to S7 References
