# Supplementary material for: On-chip distribution of quantum information using traveling phonons
Source: Sci Adv. 2022 Nov 18;8(46):eadd2811. doi: 10.1126/sciadv.add2811 (PMC9674299; doi:10.1126/sciadv.add2811)
Supplement: Supplementary file 1 — Sections S1 to S7 Figs. S1 to S7 References [file sciadv.add2811_sm.pdf]

Science Advances
AAAS

Supplementary Materials for

On-chip distribution of quantum information using traveling phonons

Amirparsa Zivari *et al.*

Corresponding author: Simon Gröblacher, s.groeblacher@tudelft.nl

Sci. Adv. **8**, eadd2811 (2022)
DOI: 10.1126/sciadv.add2811

This PDF file includes:

Sections S1 to S7
Figs. S1 to S7
References

1. *Design*

Our device, which is shown in Fig. 1a in the main text, is composed of three distinct parts: a mirror, a cavity and a waveguide. To design the device we use finite element simulations (COMSOL) and engineer a suspended silicon nanobeam (width 529 nm and thickness of 250 nm) with elliptical holes patterned into it. The hole dimensions are varied along the beam in order to realize the different parts of our device. The finite element simulation of the full structure is shown in Fig. S1a. The left part (blue) is a phononic and photonic mirror, with the respective bandgaps at the resonance frequencies of the cavity. The optomechanical cavity, acting as the source and detector for phonons, has a co-localized single optical mode in the telecom band and a single mechanical mode around 5 GHz, similar to [36,44]. The phononic waveguide (green) is a single mode phononic waveguide for the symmetric mechanical breathing mode in the frequency range of interest (around 5 GHz). Only this mode is considered, as it matches the mechanical mode shape of the cavity, enabling large mechanical coupling between the cavity and the waveguide. The first part of the waveguide, referred to as the "lead waveguide" in the figure, acts as a photonic mirror having a bandgap in the telecom range. The unit cell of this part, together with its optical and mechanical band structure, are shown in Fig. S1b and c, respectively. As a result of our design, the optical mode stays confined inside the optomechanical cavity, while the mechanical mode is guided with very little loss through the waveguide, as shown in Fig. S1a. The second part of the waveguide is connected to the substrate via phononic shield clamps for structural support. The band diagram of the phononic shield together with the unit cell are shown in Fig. S1d and e. The shield minimizes the mechanical loss from the waveguide to the substrate, fully confining the mechanical mode in the waveguide. The design of the waveguide with clamping differs from the lead waveguide to avoid perturbations of its band structure. The unit cell shape of this waveguide and the band structure are shown in Fig. S1f and g. Note how the mode in the waveguide has an approximately linear dispersion in the range of interest.

2. *Mechanical Lifetime*

To measure the mechanical lifetime of the device (T_1) we send a series of red-detuned double pulses with the interferometer delay arm open. The strong first pulse creates, via optical absorption, a relatively large thermal population that is probed by the second pulse, and which is delayed by time Δt_{ro}. With this pump-probe experiment we can access the uncalibrated thermal population of the device in time [39] (see Fig. S2). From the exponential decay we measure $T_1 \approx 2.2\,\mu s$, much longer than the 126 ns delay used in the experiments. We set the time between trials in all experiments equal to 15 µs, to let the population fully decay. Note that while the device is intentionally designed to have a short lifetime in order to allow for a high repetition rate of the experiment, previous work with similar structures has reported lifetime up to 5.5 ms [33]. We would also like to note that an additional phononic shield period at the mirror side (blue part in Fig. 1a in the main text) does not increase the lifetime any further. The increase in thermal population for short delays ($\Delta t_{ro} < 1\,\mu s$) is given by the delayed absorption [36].

3. *Thermal occupancy of the mechanical mode*

In order to determine the thermal occupation of the mode of interest we send trains of alternating write and read pulses to the device, as shown in Fig. S3a. From the asymmetry in clickrates of these pulses we can calculate the thermal mechanical population n_{th}. We adjust the scattering probability by sweeping the energy of the pulses and measure the resulting thermal occupation, see Fig. S3b. To further mimic the same heating conditions as in the experiment, without the optomechanically excited coherent population created from the write pulses, we use heating pre-pulses from the read laser. The alternating pulses, used to measure n_{th}, are sent at a delay

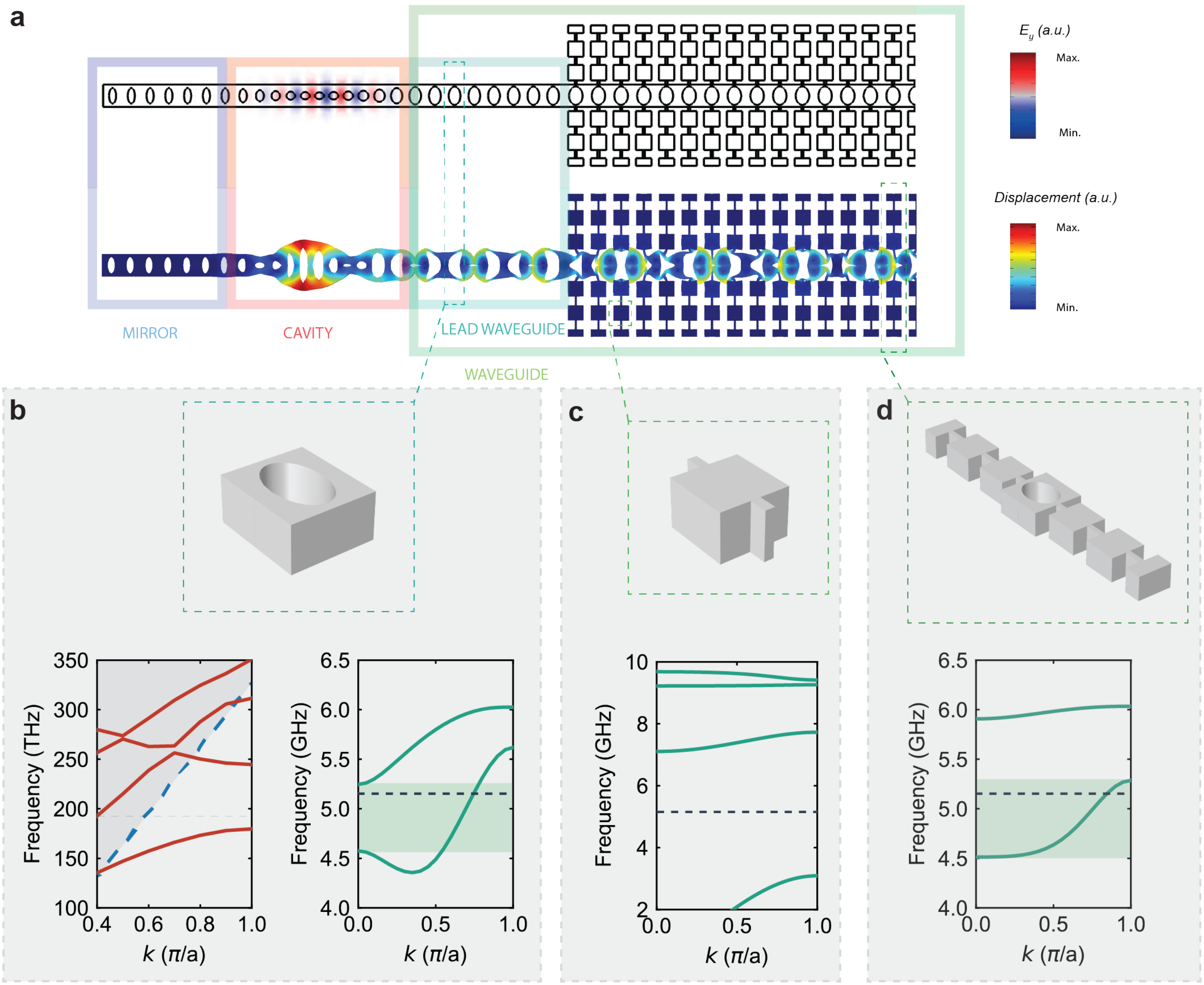

FIG. S1. **Device design.** a) Optical (top) and mechanical (bottom) eigenmode simulations of the full structure. The mechanical mode shown is a cavity-waveguide supermode. Note how the optical mode is confined in the cavity, while the mechanical mode is extended into the waveguide. b) Lead waveguide unit cell, with its optical (left) and mechanical (right) band diagram shown below. The gray area for the optical part depicts the light cone, delimited by the blue dashed line. c) Shield unit cell, used in the clamps to connect the waveguide to the substrate and its mechanical band diagram below. d) Unit cell of the shield-clamped waveguide, with its band structure shown underneath. In all plots the horizontal black dashed lines are the working optical and mechanical frequencies, while the highlighted area are the single mode regions of the waveguides.

given by the round-trip time τ and have a fixed energy, while the energy of the pre-pulse is swept. In Fig. S3 we show the pulse scheme for these measurements, as well as the thermal occupation at the second pulse (in the experiment the read pulse) as a function of the scattering probability of the first pulse (in the experiment the write pulse). We use the two asymmetry measurements (Fig. S3b and d) to choose the single write/read scattering probabilities that will give a total thermal population below 0.1, with a third of this thermal occupation given by the first pulse and the rest by the second (to minimize the effects of delayed heating).

We then measure the values of n_{th} for the pulses used in the experiments. We send heating pre-pulses and use the alternating pulses to measure the thermal population, as drawn in Fig. S3e. Each of these pulses has the corresponding energy used in the experiments and all are delayed by $\tau/2$ with respect to each other. In Fig. S3f we report the measured thermal occupation for the four pulses of the experiments, for the three sets of scattering probabilities used: in blue the one for the additional phononic entanglement data and the Bell test (Fig. 4 in the main text), in orange for the double pulse cross correlation and the phononic entanglement with the

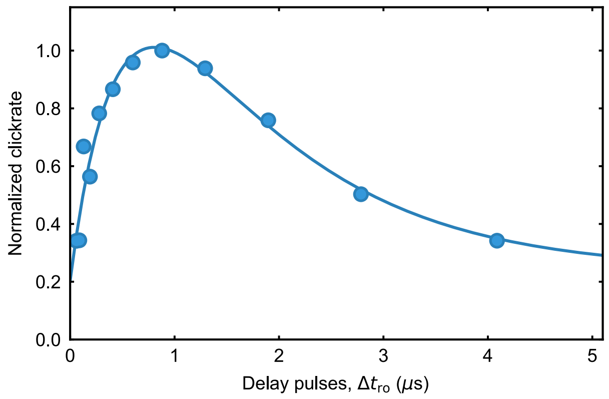

FIG. S2. **Mechanical lifetime.** Normalized clickrates from the probe pulse that gives an uncalibrated measure of the thermal population in time. The delay between pump and probe pulse is Δt_{ro}. We extract $T_1 \approx 2.2\,\mu$s, by fitting the data (solid curve).

sweep in ϕ_{w} (Fig. 3 in the main text) and in green for the phase calibration (section 6). Note how the thermal population increases non-linearly with increasing number of pulses due to delayed heating, as is clearly visible in Fig. S3.

4. Experimental setup

A sketch of the experimental setup is shown in Fig. S4. Two continuous-wave (CW) lasers (write and read) are frequency-locked to one another by detecting the interference between their light on a fast detector (in particular: the light from the write laser and the second order sideband of the read laser generated by EOM 2, which have a frequency difference of 2×110 MHz). The laser light from the CW lasers is filtered to remove GHz noise using fiber filters. The pulses are created by gating the CW light with 110 MHz AOMs, which are driven by an arbitrary waveform generator and the laser pulses are combined on a beam splitter. A phase EOM, driven by another AWG, is used to add a phase offset to the "Late" pulses (to set ϕ_{r} and ϕ_{w}). The pulses are then routed to a circulator and to the cryostat, where a lensed fiber allows the coupling to the device's optical waveguide, with efficiency of $\eta_{\mathrm{c}} \approx 50\%$. The light from the device is fed to an unbalanced Mach-Zehnder interferometer, defined by BS 1 and BS 2, where the time delay between the arms is $\tau/2 = 63$ ns. These two BSs have a relative difference in the splitting ratio of the two output ports smaller than 0.5%, while the losses in both lines are negligible. The interferometer is actively stabilized using a home-built fiber stretcher controlled by a PID loop that uses the signal from pulses coming from the INT LOCK LINE (see section 5 for more details). The polarization of the two arms are matched at BS 3 using the fiber polarization controller 1 (FPC 1). The light from the interferometer is filtered by two sets of free space optical Fabry-Pérot cavities (F 1 and F 2) with suppression ratios of the strong control pulses of about 115 dB (F 1) and 112 dB (F 2). This gives a pump pulse leakage rate of 2×10^{-7} and 4×10^{-7} photons per repetition from the write pulse and 1.4×10^{-6} and $2.6\times10{-}6$ from the read pulse, for F 1 and F 2, respectively. Note that the two sets of filters have a CW efficiency of transmission at resonance of $\sim$65%. Due to the different total bandwidth of 40 MHz (80 MHz) for F 1 (F 2), the relative transmission efficiency of the pulses is about 40% lower for F 1 using 30 ns long pulses. The experiment is paused and the filters are locked on resonance with the cavity every 8 s, flipping the switches to use the CW signal from the FILTER LOCK LINE (detectors not shown). The average time needed to lock the two filter setups is about 1 s. The signal photons are detected using superconducting nanowire single photon detectors (SNSPD).

5. Phase stability

A fundamental part of the experiment is the phase difference acquired by the pulses in the unbalanced Mach-Zehnder interferometer, which has to be actively locked. In order to do so, the two strong control pulses from the read laser line are routed via a 90:10 BS to a long delay line ($\approx$1 µs of delay) to have them temporally spaced from the signal. A PBS is inserted in the line to minimize polarization drifts. After passing through the unbalanced interferometer, the pulses are reflected by the first cavity of both filter setups and are routed by circulators to a balanced detector. A sample&hold board (not shown in Fig S4) is used to select the correct pulse and feed it as the input voltage to a Red Pitaya board. The output of the board is amplified (not shown) and sent to the fiber stretcher. The Red Pitaya runs a PID program [45] and the feedback loop is ultimately limited by the bandwidth of the fiber stretcher (approx. 20 kHz).

The phase stability can be measured by tracking the voltage of the locking pulse on the balanced detector. In Fig. S5a (b) we plot the occurrence histogram of the phase difference acquired passing the interferometer for the write (read) pulses, in the case the interferometer is locked (in blue (red)), or unlocked (in green). The FWHM are $\approx \pi/7$ for the write and $\approx \pi/20$ for the read pulses. This phase spread is the same for the Stokes (anti-Stokes) scattered photons. Note that the FWHM of phase difference for write and read pulses are different, since the phase acquired by the write laser pulse also depends on the relative frequency jitter of the two lasers. This will only affect the pulses from the write laser since the lock pulses are generated from the read laser. This frequency jitter is reported in Fig. S5c, where the oc-

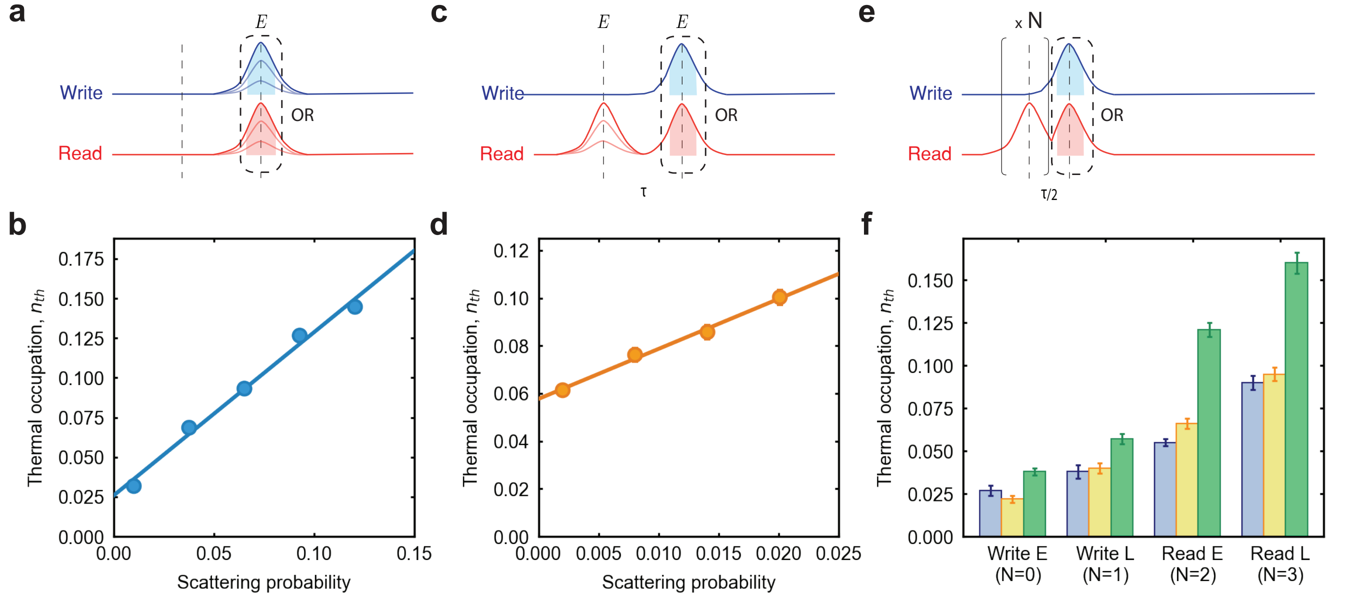

FIG. S3. **Thermal occupation measurements.** a) Pulse scheme for the thermal occupation measurement. We send trains of pulses alternating between write and read to measure the asymmetry in their scattering probabilities. The opaque pulses represents the sweep in energy of the pulse, while the shaded area is the integration region. b) Thermal occupation as a function of the measured scattering probability for a single pulse (equivalent to the read "Early" situation in the experiments). The solid line is a linear fit to the data. c) Same as a) for the two pulse calibration of the thermal occupation. Note how the read pre-pulse is only for heating the mechanical mode and its energy is swept. The alternating pulses used to measure the $n_{\rm th}$ are in the black dashed box and have fixed energy. d) Thermal occupation of the second pulse (read "Early" in the experiment) as a function of the measured scattering probability of the first pulse (write "Early" in the experiment). Note how the offset in this measurement strongly depends on the energy of the second pulse, which in this case is 225 fJ (twice the energy of the single read pulses used in the phononic entanglement experiment of Fig. 3 in the main text). The solid line is a linear fit to the data. e) Same as a) but for multiple pre-pulses. We send N pre-pulses (with $N = (0, 1, 2, 3)$ for write E, write L, read E and read L, respectively) and use the alternating pulses in the dashed black box to measure the thermal population. Each pulse has the energy used in the experiments. f) Measured thermal occupation for the four pulses used in the experiments. In blue for the scattering probabilities used in the measurements for the additional phononic entanglement data and Bell test (Fig. 4 in the main text), in orange for the double pulse cross correlation and the phononic entanglement with the sweep in $\phi_{\rm w}$ (Fig. 3 in the main text) and in green for the phase calibration (section 6). All error bars are one standard deviation.

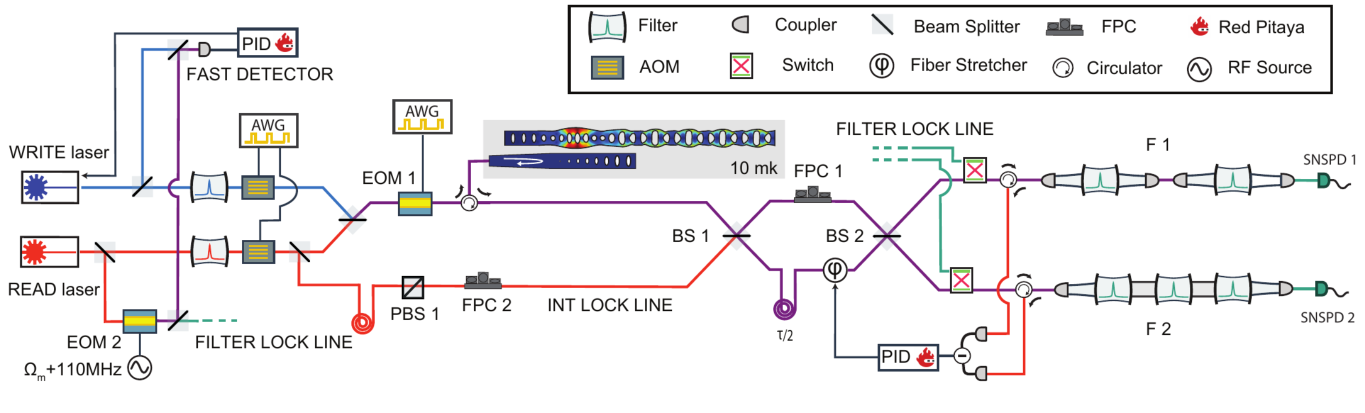

FIG. S4. **Experimental scheme.** Detailed scheme of the setup (see text for more details). AOM are the acousto-optic modulators, AWG the arbitrary waveform generators, EOM the electro-optic modulator, VOA the variable optical attenuator, BS the beamsplitters, and PBS the polarizing BS, FPC the fiber polarization controller and SNSPD the superconducting nanowire single-photon detectors. $\Omega_{\rm m}$ the mechanical frequency. All the components (apart from the free-space filters F 1 and F 2) are fiber based.

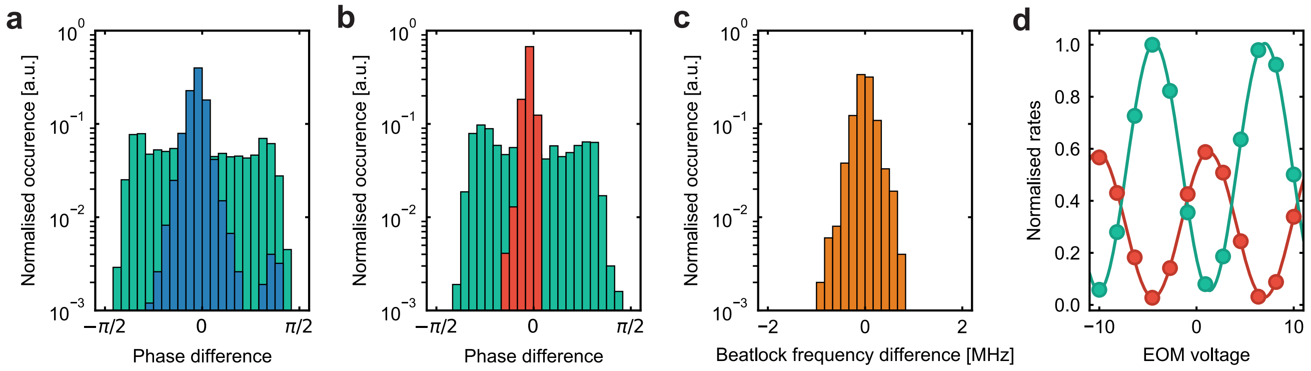

FIG. S5. **Setup characterization.** a) Occurrence histogram of the phase difference acquired by the write pulses when the interferometer is locked (in blue) and unlocked (in green). The FWHM is $\approx \pi/7$. b) Same as a) for the read pulses, for the case of a locked interferometer (in red) and unlocked (in green). The FWHM here is $\approx \pi/20$. c) Occurrence histogram of the frequency difference of the write and read lasers as from the beatlock. Here the FWHM is $\approx 0.5\,$MHz. This relative frequency jitter causes a bigger phase difference spread for the write pulses compared to the read ones. d) Normalized count rates of SNSPD1 (red) and SNSPD2 (green) for weak coherent pulses on resonance with the filter cavities. Sweeping the EOM voltage results in the observed interference pattern, which we use to calibrate the phase given by the EOM. In this case the interferometer visibility is $V_{\text{int}} \approx 94\,\%$. Error bars are one standard deviation and are too small to be seen.

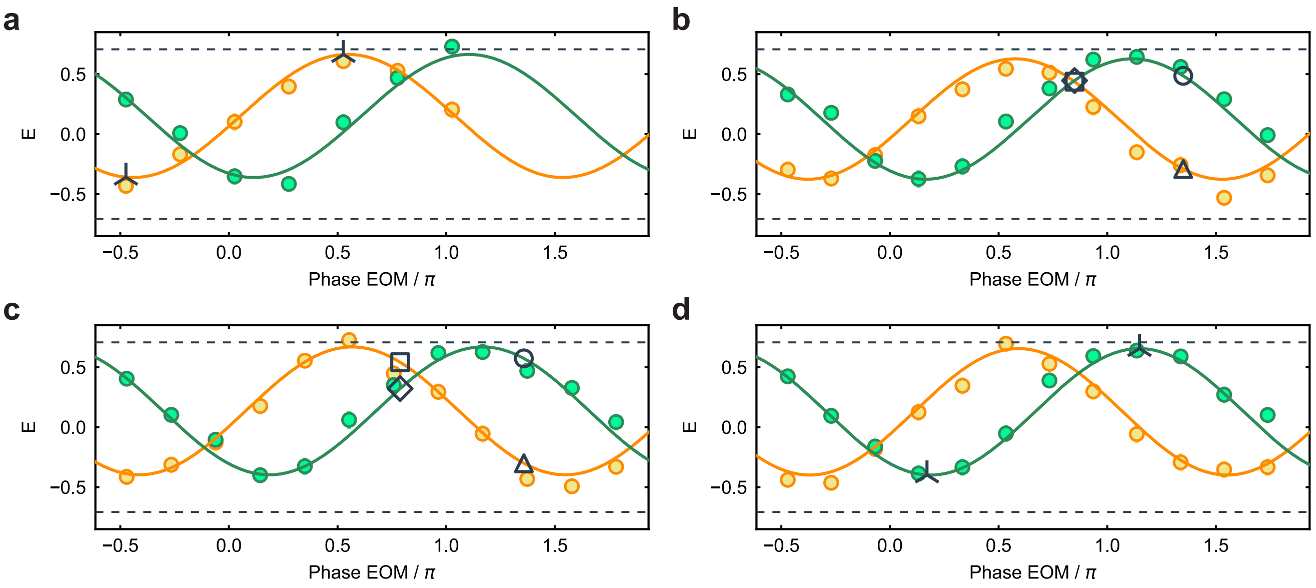

FIG. S6. **Bell measurements.** a) Correlation coefficients E, sweeping ϕ_{w} and for $\phi_{\mathrm{r}} = 0$ (in orange) and $\phi_{\mathrm{r}} = \pi/2$ (in green), for higher pulse energies with respect to the main text. The black markers (tri) are at the chosen phases for the additional measure of R. b), c) Same as a) for the calibration of the phases for the Bell test. The chosen points differ from the optimal theoretical points of $\phi_0 \pm \pi/4$ by no more than $\approx \pi/20$. The black markers are square, triangle, diamond and circle for the CHSH point $(\phi_{\mathrm{w}}^0, \phi_{\mathrm{r}}^0)$, $(\phi_{\mathrm{w}}^1, \phi_{\mathrm{r}}^0)$, $(\phi_{\mathrm{w}}^0, \phi_{\mathrm{r}}^1)$ and $(\phi_{\mathrm{w}}^1, \phi_{\mathrm{r}}^1)$ respectively. d) As reported in a) for the additional measurement of R with $\phi_{\mathrm{r}} = \pi/2$. All errors are one standard deviation and are too small to be seen. The small asymmetry in the value of E around zero is a result of the different filter setup efficiencies (see section 4).

currence histogram of the frequency difference from the beatlock is shown. Here the FWHM is around 0.5 MHz.

We use FPC 2 to balance the lock signal from the INT LOCK LINE at the balanced detector. We then lock the interferometer and use the first order interference from very weak pulses from the write laser, on resonance with the filter cavities, to measure the interferomenter visibility. We set the EOM voltage to the maximum visibility point and maximize it using FPC 1 (i.e. we align the polarization of the signal) while compensating with FPC

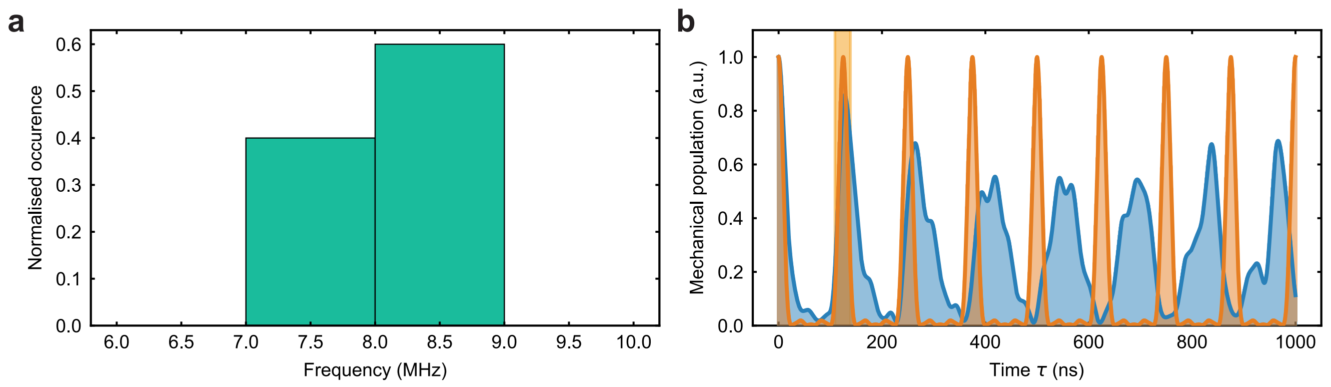

FIG. S7. **Mechanical free-spectral range.** a) Normalized histogram of the FSR between the mechanical peaks shown in Fig. 2c of the main text with mean value of 8.3 MHz and standard deviation 0.8 MHz. b) The numerically calculated time-domain mechanical population (normalized) for the device spectrum (blue) and ideal spectrum with constant FSR (orange). The shaded area is the same as in Fig. 2c.

2 for the lock pulses. A typical interference pattern is shown in Fig. S5d. We report an average interferometer visibility of $V_{\mathrm{int}} \approx 94\,\%$ during the whole experiment.

6. *Phase calibration*

To perform the measurements in the main text we need to first accurately calibrate the phase setting. This is done by performing a measurement of E while sweeping ϕ_{w} and for two settings of ϕ_{r}. Here we use higher pulse energies with respect to the actual measurement reported in the main text, such that the scattering probability increases at the expense of having a lower value of E. We use 90 fJ (225 fJ)) for the write (read) pulses, which gives $p_{\mathrm{w}} = 0.6\%$, $p_{\mathrm{r}} = 1.4\%$. With these settings we obtain more than 200 events per point in about 30 minutes. Fig. S6 shows several such calibration measurements for:

- the additional measurement of the R value (Fig. S6a),
- the two runs of integration for the Bell test (Fig. S6b, c)
- and for a final measure of R with $\phi_{\mathrm{r}} = \pi/2$ (and to check that the phase difference during the second run of integration for the Bell test is small, Fig. S6d).

The values in orange are for $\phi_{\mathrm{r}} = 0$, while $\phi_{\mathrm{r}} \approx \pi/2$ is shown in green. A small deviation from the desired $\pi/2$ phase difference between the orange and green curves can be seen and the exact values are $\phi_{\mathrm{r}} = \pi/1.8, \pi/1.9, \pi/1.7, \pi/1.8$, for Fig. S6a,b,c,d respectively. For the fits of the two datasets we use a sinusoidal function, which serves as a guide to the eye and to numerically calculate the optimal phase points. For the additional measure of R we simply use the phases where E is maximum and minimum (tri markers, Fig. S6a and d). For the Bell test, instead, we use the fit to numerically calculate the expected S value and choose the phases where the expected S is maximum (square, triangle, diamond and circle markers for the CHSH point $(\phi^0_{\mathrm{w}}, \phi^0_{\mathrm{r}})$, $(\phi^1_{\mathrm{w}}, \phi^0_{\mathrm{r}})$, $(\phi^0_{\mathrm{w}}, \phi^1_{\mathrm{r}})$ and $(\phi^1_{\mathrm{w}}, \phi^1_{\mathrm{r}})$ respectively). In doing so, the experimentally obtained values differ slightly from the theoretical optimal point of $\phi_0 - \pi/4$ and $\phi_0 + \pi/4$ by a small margin ε. The value for ε for the data in Fig. S6b (c) is $\approx \pi/30$ and $\approx \pi/20$ ($\approx -\pi/40$ and $\approx \pi/20$), respectively. The phase offset ϕ_{off} is calibrated using the maximum and minimum point of E. We choose this particular calibration method to compensate for eventual drifts in the phase offset (ϕ_{off}), as well as small inaccuracies of phase difference for two sets of measurements with different ϕ_{r}. Using light to lock the interferometer at a different frequency and from a different path from that of the signal, gives rise to a (fixed) phase offset ϕ_{off} in the entangled state (see section 4). Note that without an external reference PBS a relative change in the polarization between lock pulses and signal pulses will cause a change in the phase shift ϕ_{off}. However, in our case, the relative change in ϕ_0 (equivalently for ϕ_{off}) is less than $\pi/50$ in all four measurements.

To further avoid that phase drifts affect only parts of the datasets, we integrate for one hour at each phase point at a time. We then cycle the chosen phases 4 times for phononic entanglement data (and 12 for the longer integration points), and 16 times for the Bell test.

7. *Effect of non-constant FSR*

The small dispersion in the waveguide causes a non-constant FSR between the mechanical peaks (see Fig. 2b

in the main text). In Fig. S7a we report the histogram of the FSR between the mechanical modes. By using the frequency and amplitude of each mechanical peak in the spectrum, we numerically simulate the time-domain behavior of the mechanical system and compare it with the simulation of the ideal case (i.e. with perfectly constant FSR) [33]. As can be seen in Fig. S7b (blue graph), the mechanical packet is broadened and dimmer after several round-trips due to dispersion of the waveguide compared to the ideal case (orange graph). In this calculation the energy decay of the phonons has not been considered, tracing out any mechanical dissipation, and thus only taking the effect of the dispersion and non-constant FSR into account.

REFERENCES AND NOTES

1. F. Arute, K. Arya, R. Babbush, D. Bacon, J. C. Bardin, R. Barends, R. Biswas, S. Boixo, F. G. S. L. Brandao, D. A. Buell, B. Burkett, Y. Chen, Z. Chen, B. Chiaro, R. Collins, W. Courtney, A. Dunsworth, E. Farhi, B. Foxen, A. Fowler, C. Gidney, M. Giustina, R. Graff, K. Guerin, S. Habegger, M. P. Harrigan, M. J. Hartmann, A. Ho, M. Hoffmann, T. Huang, T. S. Humble, S. V. Isakov, E. Jeffrey, Z. Jiang, D. Kafri, K. Kechedzhi, J. Kelly, P. V. Klimov, S. Knysh, A. Korotkov, F. Kostritsa, D. Landhuis, M. Lindmark, E. Lucero, D. Lyakh, S. Mandrà, Jarrod R. Mc Clean, M. M. Ewen, A. Megrant, X. Mi, K. Michielsen, M. Mohseni, J. Mutus, O. Naaman, M. Neeley, C. Neill, M. Y. Niu, E. Ostby, A. Petukhov, J. C. Platt, C. Quintana, E. G. Rieffel, P. Roushan, N. C. Rubin, D. Sank, K. J. Satzinger, V. Smelyanskiy, K. J. Sung, M. D. Trevithick, A. Vainsencher, B. Villalonga, T. White, Z. Jamie Yao, P. Yeh, A. Zalcman, H. Neven, J. M. Martinis, Quantum supremacy using a programmable superconducting processor. *Nature* **574**, 505–510 (2019).

2. C. Zhong, Z. Wang, C. Zou, M. Zhang, X. Han, W. Fu, M. Xu, S. Shankar, M. H. Devoret, H. X. Tang, L. Jiang, Proposal for heralded generation and detection of entangled microwave–optical-photon pairs. *Phys. Rev. Lett.* **124**, 010511 (2020).

3. H. J. Kimble, The quantum internet. *Nature* **453**, 1023–1030 (2008).

4. N. Sangouard, C. Simon, H. de Riedmatten, N. Gisin, Quantum repeaters based on atomic ensembles and linear optics. *Rev. Mod. Phys.* **83**, 33–80 (2011).

5. L. M. K. Vandersypen, H. Bluhm, J. S. Clarke, A. S. Dzurak, R. Ishihara, A. Morello, D. J. Reilly, L. R. Schreiber, M. Veldhorst, Interfacing spin qubits in quantum dots and donors—Hot, dense, and coherent. *npj Quantum Inf.* **3**, 34 (2017).

6. M. Wallquist, K. Hammerer, P. Rabl, M. Lukin, P. Zoller, Hybrid quantum devices and quantum engineering. *Phys. Scr.* **2009**, 014001 (2009).

7. J. T. Hill, A. H. Safavi-Naeini, J. Chan, O. Painter, Coherent optical wavelength conversion via cavity optomechanics. *Nat. Commun.* **3**, 1196 (2012).

8. A. Vainsencher, K. J. Satzinger, G. A. Peairs, A. N. Cleland, Bi-directional conversion between microwave and optical frequencies in a piezoelectric optomechanical device. *Appl. Phys. Lett.* **109**, 033107 (2016).

9. R. W. Andrews, R. W. Peterson, T. P. Purdy, K. Cicak, R. W. Simmonds, C. A. Regal, K. W. Lehnert, Bidirectional and efficient conversion between microwave and optical light. *Nat. Phys.* **10**, 321–326 (2014).

10. X. Han, W. Fu, C. Zhong, C.-L. Zou, Y. Xu, A. A. Sayem, M. Xu, S. Wang, R. Cheng, L. Jiang, H. X. Tang, Cavity piezo-mechanics for superconducting-nanophotonic quantum interface. *Nat. Commun.* **11**, 3237 (2020).

11. G. Arnold, M. Wulf, S. Barzanjeh, E. S. Redchenko, A. Rueda, W. J. Hease, F. Hassani, J. M. Fink, Converting microwave and telecom photons with a silicon photonic nanomechanical interface. *Nat. Commun.* **11**, 4460 (2020).

12. W. Jiang, C. J. Sarabalis, Y. D. Dahmani, R. N. Patel, F. M. Mayor, T. P. McKenna, R. Van Laer, A. H. Safavi-Naeini, Efficient bidirectional piezo-optomechanical transduction between microwave and optical frequency. *Nat. Commun.* **11**, 1166 (2020).

13. M. Forsch, R. Stockill, A. Wallucks, I. Marinković, C. Gärtner, R. A. Norte, F. van Otten, A. Fiore, K. Srinivasan, S. Gröblacher, Microwave-to-optics conversion using a mechanical oscillator in its quantum groundstate. *Nat. Phys.* **16**, 69 (2020).

14. R. Stockill, M. Forsch, F. Hijazi, G. Beaudoin, K. Pantzas, I. Sagnes, R. Braive, S. Gröblacher, Ultra-low-noise microwave to optics conversion in gallium phosphide, arXiv:2107.04433 (2021).

15. M. Mirhosseini, A. Sipahigil, M. Kalaee, O. Painter, Superconducting qubit to optical photon transduction. *Nature* **588**, 599–603 (2020).

16. T. Palomaki, J. Teufel, R. Simmonds, K. Lehnert, Entangling mechanical motion with microwave fields. *Science* **342**, 710–713 (2013).

17. Y. Chu, P. Kharel, W. H. Renninger, L. D. Burkhart, L. Frunzio, P. T. Rakich, R. J. Schoelkopf, Quantum acoustics with superconducting qubits. *Science* **358**, 199–202 (2017).

18. R. Blatt, D. Wineland, Entangled states of trapped atomic ions. *Nature* **453**, 1008–1015 (2008).

19. P. Delsing, A. N. Cleland, M. J. A. Schuetz, J. Knörzer, G. Giedke, J Ignacio Cirac, K. Srinivasan, M. Wu, K. C. Balram, C. Bäuerle, T. Meunier, C. J. B. Ford, P. V. Santos, E. Cerda-Méndez, H. Wang, H. J. Krenner, E. D. S. Nysten, M. Weiß, G. R. Nash, L. Thevenard, C. Gourdon, P. Rovillain, M. Marangolo, J.-Y. Duquesne, G. Fischerauer, W. Ruile, A. Reiner, B. Paschke, D. Denysenko, D. Volkmer, A. Wixforth, H. Bruus, M. Wiklund, J. Reboud, J. M. Cooper, Y. Q. Fu, M. S. Brugger, F. Rehfeldt, C. Westerhausen, The 2019 surface acoustic waves roadmap. *J. Phys. D Appl. Phys.* **52**, 353001 (2019).

20. D. A. Golter, T. Oo, M. Amezcua, I. Lekavicius, K. A. Stewart, H. Wang, Coupling a surface acoustic wave to an electron spin in diamond via a dark state. *Phys. Rev. X* **6**, 041060 (2016).

21. A. Bienfait, K. J. Satzinger, Y. P. Zhong, H.-S. Chang, M.-H. Chou, C. R. Conner, É. Dumur, J. Grebel, G. A. Peairs, R. G. Povey, A. N. Cleland, Phonon-mediated quantum state transfer and remote qubit entanglement. *Science* **364**, 368–371 (2019).

22. R. P. G. McNeil, M. Kataoka, C. J. B. Ford, C. H. W. Barnes, D. Anderson, G. A. C. Jones, I. Farrer, D. A. Ritchie, On-demand single-electron transfer between distant quantum dots. *Nature* **477**, 439–442 (2011).

23. S. Hermelin, S. Takada, M. Yamamoto, S. Tarucha, A. D. Wieck, L. Saminadayar, C. Bäuerle, T. Meunier, Electrons surfing on a sound wave as a platform for quantum optics with flying electrons. *Nature* **477**, 435–438 (2011).

24. T. Neuman, M. Eichenfield, M. E. Trusheim, L. Hackett, P. Narang, D. Englund, A phononic interface between a superconducting quantum processor and quantum networked spin memories. *npj Quantum Inf.* **7**, 121 (2021).

25. M. Aspelmeyer, T. J. Kippenberg, F. Marquardt, Cavity optomechanics. *Rev. Mod. Phys.* **86**, 1391 (2014).

26. S. Barzanjeh, A. Xuereb, S. Gröblacher, M. Paternostro, C. A. Regal, and E. M. Weig, Optomechanics for quantum technologies. *Nat. Phys.* **18**, 15–24 (2022).

27. M. K. Ekström, T. Aref, J. Runeson, J. Björck, I. Boström, P. Delsing, Surface acoustic wave unidirectional transducers for quantum applications. *Appl. Phys. Lett.* **110**, 073105 (2017).

28. É. Dumur, K. J. Satzinger, G. A. Peairs, M.-H. Chou, A. Bienfait, H.-S. Chang, C. R. Conner, J. Grebel, R. G. Povey, Y. P. Zhong, A. N. Cleland, Quantum communication with itinerant surface acoustic wave phonons. *npj Quantum Inf.* **7**, 173 (2021).

29. A. Bienfait, Y. P. Zhong, H.-S. Chang, M.-H. Chou, C. R. Conner, É. Dumur, J. Grebel, G. A. Peairs, R. G. Povey, K. J. Satzinger, A. N. Cleland, Quantum erasure using entangled surface acoustic phonons. *Phys. Rev. X* **10**, 21055 (2020).

30. J. Chan, A. H. Safavi-Naeini, J. T. Hill, S. Meenehan, O. Painter, Optimized optomechanical crystal cavity with acoustic radiation shield. *App. Phys. Lett.* **101**, 081115 (2012).

31. A. Wallucks, I. Marinković, B. Hensen, R. Stockill, S. Gröblacher, A quantum memory at telecom wavelengths. *Nat. Phys.* **16**, 772–777 (2020).

32. R. N. Patel, Z. Wang, W. Jiang, C. J. Sarabalis, J. T. Hill, A. H. Safavi-Naeini, Single-mode phononic wire. *Phys. Rev. Lett.* **121**, 040501 (2018).

33. A. Zivari, R. Stockill, N. Fiaschi, S. Gröblacher, Non-classical mechanical states guided in a phononic waveguide. *Nat. Phys.* **18**, 789–793 (2022).

34. J. F. Clauser, M. A. Horne, A. Shimony, R. A. Holt, Proposed experiment to test local hidden-variable theories. *Phys. Rev. Lett.* **23**, 880 (1969).

35. I. Marinković, A. Wallucks, R. Riedinger, S. Hong, M. Aspelmeyer, S. Gröblacher, An optomechanical Bell test. *Phys. Rev. Lett.* **121**, 220404 (2018).

36. R. Riedinger, S. Hong, R. A. Norte, J. A. Slater, J. Shang, A. G. Krause, V. Anant, M. Aspelmeyer, S. Gröblacher, Non-classical correlations between single photons and phonons from a mechanical oscillator. *Nature* **530**, 313–316 (2016).

37. S. Weis, R. Rivière, S. Deléglise, E. Gavartin, O. Arcizet, A. Schliesser, T. J. Kippenberg, Optomechanically induced transparency, *Science* **330**, 1520–1523 (2010).

38. R. Stockill, M. Forsch, G. Beaudoin, K. Pantzas, I. Sagnes, R. Braive, S. Gröblacher, Gallium phosphide as a piezoelectric platform for quantum optomechanics. *Phys. Rev. Lett.* **123**, 163602 (2019).

39. S. Hong, R. Riedinger, I. Marinković, A. Wallucks, S. G. Hofer, R. A. Norte, M. Aspelmeyer, S. Gröblacher, Hanbury Brown and Twiss interferometry of single phonons from an optomechanical resonator. *Science* **358**, 203–206 (2017).

40. K. Børkje, A. Nunnenkamp, S. M. Girvin, Proposal for entangling remote micromechanical oscillators via optical measurements. *Phys. Rev. Lett.* **107**, 123601 (2011).

41. R. Riedinger, A. Wallucks, I. Marinković, C. Löschnauer, M. Aspelmeyer, S. Hong, S. Gröblacher, Remote quantum entanglement between two micromechanical oscillators. *Nature* **556**, 473–477 (2018).

42. L. M. Duan, M. D. Lukin, J. I. Cirac, P. Zoller, Long-distance quantum communication with atomic ensembles and linear optics. *Nature* **414**, 413–418 (2001).

43. J. C. Taylor, E. Chatterjee, W. F. Kindel, D. Soh, M. Eichenfield, Reconfigurable quantum phononic circuits via piezo-acoustomechanical interactions. *npj Quantum Inf.* **8**, 19 (2022).

44. J. Chan, T. P. M. Alegre, A. H. Safavi-Naeini, J. T. Hill, A. Krause, S. Gröblacher, M. Aspelmeyer, O. Painter, Laser cooling of a nanomechanical oscillator into its quantum ground state. *Nature* **478**, 89–92 (2011).

45. M. A. Luda, M. Drechsler, C. T. Schmiegelow, J. Codnia, Compact embedded device for lock-in measurements and experiment active control. *Rev. Sci. Instr.* **90**, 023106 (2019).
